# End of life care for frail older patients in family practice (ELFOP) – protocol of a longitudinal qualitative study on needs, appropriateness and utilisation of services

**DOI:** 10.1186/1471-2296-14-52

**Published:** 2013-05-03

**Authors:** Gabriele Müller-Mundt, Jutta Bleidorn, Karin Geiger, Katharina Klindtworth, Sabine Pleschberger, Eva Hummers-Pradier, Nils Schneider

**Affiliations:** 1 Institute for Epidemiology, Social Medicine and Health Systems Research, Hannover Medical School, Carl-Neuberg-Straße 1, Hannover 30625, Germany; 2 Institute for General Practice, Hannover Medical School (MHH), Carl-Neuberg-Straße 1, Hannover 30625, Germany; 3 Institute of Nursing and Care Sciences Research, Department of Nursing Science and Gerontology, The Health and Life Sciences University (UMIT) Vienna, Opernring 5, Vienna, A, 1010, Austria; 4 Department of General Practice, University Medical Centre Göttingen (UMG), Humboldtallee 38, Göttingen, D, 37073, Germany

**Keywords:** Frailty, Elderly patients, Informal caregivers, Family medicine, End-of-life care, Palliative care, Longitudinal qualitative study, Multiple perspectives, Needs, Health services research

## Abstract

**Background:**

Frail elderly people represent a major patient group in family practice. Little is known about the patients’ needs, and how their needs evolve over time with increasing frailty towards the end of life. This study will address end-of-life care needs, service utilisation, and experiences of frail elderly patients and their informal caregivers, with regard to family practice. This paper aims to introduce the research protocol.

**Methods/Design:**

The study uses a multiple perspective approach qualitative design. The first study part consists of serial six-monthly in-depth interviews with 30 community-dwelling elderly patients (≥70 years) with moderate to severe frailty and their key informal caregivers, over a period of 18 months. Additionally, semi-structured interviews with the patients’ family physician will be conducted. The serial interviews will be analysed with grounded theory and narrative approaches. Special attention will be paid to the comparison of distinct views of the patients’, informal caregivers’, and family physicians’ as well as on chronological aspects. In the second study part, five focus groups with experts in family medicine, geriatrics, palliative medicine, and nursing will be conducted. Finally, the implications for family practice and health care policy will be discussed in an expert workshop.

**Discussion:**

To our knowledge, this is the first prospective, longitudinal qualitative study on the needs of elderly patients with advanced frailty towards the end of life in German family practice, which integrates the perspectives of patients, informal caregivers, family physicians and other health professionals. The study will contribute to the understanding of the clinical, psychosocial and information needs of patients and their caregivers, and of respective changes of experiences and needs along the illness/frailty trajectory including the last phase of life. It will provide an empirical basis for improving patient-centred care for this increasingly relevant target group.

## Background

Due to the demographic changes, frail older patients increasingly focus attention in family practice [1]. Frailty can be defined as a state of vulnerability to adverse outcomes. It affects about 7% of people aged 65 years and older, with increasing numbers in higher age, and is associated with symptomatic long-term disease and decline in function [2]. Severe frailty is associated with increased probability of admission to a nursing home and an increased risk of death [3]. For severely frail patients, a palliative care approach may be appropriate to relieve suffering and enhance quality of life [4].

Research on palliative care focuses on specialist palliative care and cancer patients, whereas relatively little is known about the needs of older non-cancer patients in family practitioners’ care towards the end of life [5-7]. Some explorative studies indicate the central role of family practitioners in delivering end-of-life care, but also the need for improving family practitioners’ competencies, structure of services, and communication among health professionals, patients and their informal caregivers [8-10]. Recently, improving cooperation among palliative care specialists and generalists was declared a public health priority in Germany [11].

However, so far there is only little in-depth understanding of the needs and perceptions of elderly patients with severe frailty and their change over time. Based on a multi-perspective longitudinal design, this qualitative study aims to contribute to filling this research gap.

To elucidate older patients’ and their informal caregivers’ experiences when facing increasing frailty and support needs, this study refers to the trajectory concept developed by Strauss et al. on the basis of their qualitative research on death, dying, and chronic illness [12-14]. It provides a multi-dimensional frame for the analysis of the interplay between personal experiences in the course of illness/frailty, and any attempts to manage it in daily life. The trajectory concept emphasises the active role of the patients and her/his relatives as well as of healthcare professionals involved [14].

According to the different trajectory phases we focus on unstable and downward phases, when the courses of life-limiting illness and functional capacities in older age are declining. With respect to patients’ functional decline, Lunney et al. demonstrated that approximately 85% of the severely ill and dying people follow one of the three distinct illness trajectories: (1) steady progression and usually a clear terminal phase, mostly in cancer; (2) gradual decline, with episodes of acute deterioration and some recovery, with more sudden, unexpected death, often associated with life limiting chronic illness (i.e. respiratory failure); and (3) prolonged gradual decline (i.e. frailty) [15].

The study builds upon previous research of Murray et al. on end-of-life care needs in older age and life limiting illness, such as cancer, cardiac failure, and pulmonary diseases [16,17]. The studies of Murray et al. were carried out in the UK health care system which is characterised by strong primary health care with gatekeeping by family practitioners. To our knowledge, this study will be the first prospective qualitative study relating to end-of-life care of frail elderly people featuring primary health care in Germany. In the German health care system, patients have free access to medical specialists with only very limited incentives to see a family practitioner first [18,19].

With respect to the WHO-definition of palliative care [20] and the recommendations of the European Association for Palliative Care [21] the study uses an extended definition of end-of-life care including the last days, weeks, months or years. Considering end-of-life care within a wider lifespan accounts for the care needs of older people being determined not so much defined by the closeness to death but much more so by increasing frailty over a longer period of time, and the respective physical, psychological, social and spiritual consequences [22]. Therefore, this study aims to:

1. identify the clinical, psychosocial, spiritual and information needs of frail elderly patients and their informal caregivers, and the respective changes over time towards end of life,

2. describe and understand patients’ and their informal caregivers’ perception and utilisation of available services with special attention to appropriateness and access,

3. describe and understand family practitioners’ and other involved healthcare professionals’ perceptions of needs, access to and quality of services for these patients and their informal caregivers, and to

4. integrate the perceptions of patients, informal caregivers and professionals into recommendations to optimise care for frail elderly people towards the end of life.

This paper aims to introduce the research protocol of the study and to reflect upon major challenges in conducting the study.

### Ethics

The ethics committee of Hanover Medical School has approved the study in March 2012 (Registration No.: AZ 1378–2012). Written informed consent will be obtained from all participants. Consent includes the option to withdraw from the study at any time. Interviewed patients or informal caregivers might develop a personal relation to the interviewer; this represents both a benefit (i.e. finding a person who has the time to listen, and who will visit repeatedly) and a risk (expectancy of a therapeutic role, additional burden) [23]. An emergency plan for crisis intervention (i.e. psychosocial support) will be worked out for every patient with his/her family physician before the first interview.

## Methods/Design

To realise the study aims, a multi-perspective, longitudinal qualitative design was chosen referring to grounded theory [24,25] and narrative research strategies [26,27], as proposed for example by Charmaz [28]. While grounded theory is suited to capture multiple perspectives, narrative approaches hold specific potentials to unfold the sometimes unconscious and often hard-to-express thoughts, experiences and concerns of patients and their informal caregivers towards the end of life.

The study design was developed referring to strategies of serial interviewing used by Murray et al. in their research on life limiting chronic illness [29]. Longitudinal patient-centred case studies proved most suitable for exploring the evolution of complex needs and care situations in palliative and end-of-life care [17,30,31]. Compared to cross-sectional research, a longitudinal design based on serial in-depth interviews offers considerable advantages as to understanding the dynamics of illness/frailty experiences, and patients’ and their relatives’ varying needs over time. Returning for further rounds of data collection also helps to establish trust, empathy, and a deeper understanding of the frameworks people use. Furthermore, compared to cross sectional data, the longitudinal dataset broadens the analytical possibilities: data can be compared, and data can also be analysed prospectively and retrospectively from a key event, such as death [29].

### Overall study design, setting, and target groups

The *study setting* covers family practice and home care in different geographical areas of Lower Saxony, Germany, stratified to infrastructure of health care facilities (i.e. local family practitioner availability in urban/rural areas, availability of specialist palliative care teams).

**Figure 1:**
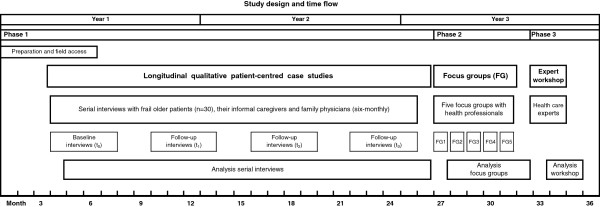
Study design and time flow.

The study design consists of three phases with the first phase constituting the main part (see Figure 1).

#### Phase 1: longitudinal patient-centred case studies

Phase 1 of the study comprises serial interviews with *community-dwelling frail older patients* (≥70 years), their main *informal caregivers* and *family physicians*[16,30].

To capture the patients’ and their caregivers’ distinct views and any changes over time, up to four in-depth interviews are conducted six-monthly within 18 months. In case a patient dies within the 18-month period, a *bereavement interview* will be performed with his/her main informal caregiver 3–6 months after the patients’ death if feasible.

The family physicians’ perspectives are particularly important since they are usually the key medical care providers for our target group. Thus, parallel to the investigation of patients’ and their caregivers’ views, semi-structured serial interviews will be conducted with the patients’ family physicians.

#### Phase 2: focus group discussions with healthcare professionals

The second phase of the study was designed to consider the patient-centred case studies in a broader context. The major findings from phase 1 will be reflected in group discussions with representatives of the main healthcare professions engaged in end-of-life care for older patients in different care settings (family physicians, geriatricians, palliative physicians, and nurse specialists in geriatric, palliative and hospice care).

#### Phase 3: expert workshop

Finally, an expert workshop with stakeholders and decision makers of health care will take place. Representatives of palliative medicine, geriatrics and family medicine, nursing, health insurance and patient organisations will discuss the key findings of the first and second phase, draw conclusions and point out opportunities for implementation of the results (i.e. with respect to teaching, education, services, process management).

### Study population and sample size

The target population of a qualitative research project must be heterogeneous, covering a large variety of patients, informal caregivers, and family physicians with different backgrounds and working conditions in different regions or different care infrastructure backgrounds, thus grasping a multitude of different perspectives and contexts. Initial field access is obtained via the network of academic teaching practices of the local Institute for General Practice at Hanover Medical School.

#### Phase 1: longitudinal patient-centred case studies

##### Patients

Community-dwelling frail older patients will be purposively recruited to reflect both sexes, and a variety of informal care arrangements as well as involvement of professional healthcare services. Frailty will be assessed with the Canadian Study of Health and Aging (CSHA) – Clinical Frailty Scale [3] and the Frailty Index SHARE FI [33,34]:

• The *CSHA-Clinical Frailty Scale* stratifies patients according to simple clinical characteristics; it captures a wide range of frailty features and shows a good performance in assessing risk of death [3].

• *SHARE FI* is a simple tool developed for the Survey of Health, Ageing and Retirement in Europe (SHARE) to explore frailty in primary care based on self-reported functional capacities and grip strength measurements [34]. Referring to Fried et al. [2], the dimensions shrinking, exhaustion, weakness, slowness, and low activity are addressed. The short instrument is applicable by non-clinicians and proved to be a robust predictor for mortality, comorbidities, and disability [34-36].

*Inclusion criteria* are moderate to severe frailty (stage 6/7 according to the CSHA-Clinical Frailty Scale) and being frail according to SHARE FI, age ≥70 years, and ability to give informed consent.

Exclusion criteria are severe dementia or an active progressive disease (i.e. cancer).

The study focuses on people living in their own homes. Participants admitted to nursing homes after the baseline interview will be followed up if they agree.

Previous qualitative longitudinal studies used sample sizes of 20–25 patients with heart failure or lung cancer [16,17,37]. Since frail elderly are a more heterogeneous target group, we calculate a slightly higher sample size of approximately 30 patients (see Table 1), which we expect to be sufficient for saturation.

**Table 1:** Calculation of the number of the serial interviews

**Interviews/Target group**	**T**_**0 **_**Baseline**	**T**_**1 **_**(6 moths)**	**T**_**2 **_**(12 months)**	**T**_**3 **_**(18 moths)**	**Total**
Serial interviews with patients/informal caregivers	30/15 = 45	25/12 = 37	19/9 = 28	12/6 = 18	**86/42 = 128**
Additional interviews with patients/informal caregivers (in the intervals)					**30**
Bereavement interviews with informal caregivers (in case of patients' death)					**7**
Serial interviews with family physicians	30	25	19	12	**86**
**Total**	**75**	**62**	**47**	**30**	**251**

We expect each participating general practitioner to recruit 1–3 patients. Based on our experiences [38], most elderly patients are willing to participate in research interviews especially if proposed by their family physician [39]. Several, repeated interviews within an 18 months period may be a barrier for some patients. However, published experiences [32,40,41] demonstrate that patients can, and indeed appreciate to talk about personal and sensitive issues such as increasing functional decline, death, dying, and bereavement in repeated interviews over a longer period of time. We expect a 30% drop out due to withdrawal or non-ability to be interviewed any more, and 30% due to death.

##### Informal caregivers

Each patient will be asked to nominate a key informal caregiver. Sampling will try to consider different gender and relationships of informal caregivers to patients (i.e. spouses, children). We anticipate that in some cases, no informal caregiver will be available, or patients do not wish to involve them, or patients wish to involve more than one informal caregiver. Thus, respecting the individual patients’ preference in some cases no informal caregiver interview will be interviewed, while in others two or more caregiver interviews will be conducted at each interview point.

##### Family physicians

Patients’ family physicians will be interviewed parallely to the patient and informal caregiver interviews. We use purposeful sampling to recruit a variety of family physicians with different profiles (men/women, single/group practice, quality of health/palliative care infrastructure in the respective geographical area).

#### Phase 2: focus group participants

Five focus group discussions with representatives of the main healthcare professions involved in end-of-life care of elderly patients will be conducted (each with 6–10 participants, reflecting a range of job experience, age, and sex). Participants will be recruited via professional organisations and regional practice networks (i.e. family practitioner network, Hannover; palliative care network in Lower Saxony). Geriatricians and nurses will be recruited by personal contacts that have successfully supported previous research [9].

##### Group 1 and 2 (family practitioners)

All family practitioners who have recruited at least one patient in phase 1 and participated in the serial interviews will be invited for the focus group discussions to share their experiences with colleagues. We assume that a total of 15–20 family practitioners will participate in the group discussions.

##### Group 3 (palliative care specialists)

Doctors and nurses working in palliative care teams are invited, as these are potential cooperation partners for family practitioners in delivering end of life care.

##### Group 4 (geriatricians)

Discussing the interview findings with geriatricians will integrate aspects of specific, active treatment and rehabilitation and thereby strengthen the wider definition of end-of-life care in this study.

##### Group 5 (nursing)

This group will primarily consist of community based nurses as these are the main cooperation partners for family practitioners in delivering primary home care for older patients.

#### Phase 3: final expert workshop

For the final expert workshop, health care stakeholders and decision makers will be invited to encompass a broad range of expertise. These are regionally active medical associations of palliative medicine, geriatrics and family medicine, nursing organisations, health insurance funds, self-government bodies (i.e. chamber of physicians, statutory organisation of sickness fund doctors, and patient organisations).

### Data collection

#### Phase 1: longitudinal patient-centred case studies

##### Serial interviews with patients and informal caregivers

The initial interviews have a strong narrative character guided through open questions as well as narrative stimuli. They intend to capture narratives focusing on the illness/frailty trajectory, as well as, for a broader scope, on people’s values and preferences for care. Furthermore, the interviews will cover patients’ and caregivers’ current concerns with regard to increasing care needs, available services and treatments, and information needs. The interview guide serves to capture the range and depth of the subjects’ experiences, while being sufficiently flexible to enable the interviewer to respond to individual patient and caregiver needs and circumstances. Basic patient-related data (i.e. diagnoses, comorbidities, recent hospital admission/s, care needs, medical aids and appliances) and the patients frailty status according to the CSHA-Clinical Frailty Scale [3] and SHARE FI [35] will be collected using a short questionnaire. In addition, SHARE FI grip strength measurements will be performed at the end of the each baseline interview, if feasible (see Table 2).

**Table 2:** Main themes of the baseline interviews with patients and informal caregivers

**Patient interview – main themes**	**Informal caregiver interview – main themes**
▪ Development of their health (problems), Illness/frailty experience	▪ Development of the patients’ and their own health (problems), illness/frailty experience
▪ Challenges and coming to terms with health problems/impairments in daily living	▪ Challenges and coming to terms with health problems/impairments in daily living
▪ Support needs	▪ Patients’ and their own support needs
▪ Perceived informal/family support	▪ Perceived informal/family support (in caring for the patients)
▪ Perceived health care/professional support	▪ Perceived health care/ professional support for the patient and themself
▪ (Social) Participation	▪ (Social) Participation of the patient and themself
▪ Future prospects, concerns, and wishes	▪ Future prospects, concerns, and wishes
▪ Biographical and social background	▪ Biographical and social background
▪ SHARE FI Questionnaire, and grip strength measurement	

The interview guide was developed referring to instruments used by our collaborators and members of our team in previous research projects [8,9,38,41] and tested for comprehensibility and handling in pilot-interviews with two home dwelling frail elderly patients and one elderly patient who had recently entered a nursing home, in spring 2012.

Between the interviews participating patients and caregivers will be phoned every 2–3 months to maintain the contact and ask about acute events or changes (i.e. nursery home admission, recent hospital admission). If appropriate, additional interviews will be arranged. We estimate an average of one additional interview per patient/informal caregiver. Furthermore, we will offer participants the opportunity to contact us at any time if there are any questions or concerns.

*Bereavement interviews with informal caregivers* will cover their own experiences and retrospective perceptions of the patients’ needs and available services in the last phase of life, as well as current problems, concerns, and needs of the bereaved. The interview guide will raise narratives of the care specifically in the last phase of life, as well as questions on how needs were met by the professional care system. Main themes addressed in the bereavement interviews are:

• development of the patients’ illness/frailty trajectory in the last months/weeks of his/her live,

• patients‘ support needs during the last months/weeks of their lifes in daily living,

• challenges and informal caregivers’ experience of the dying phase,

• informal caregivers’ support needs during the last months/weeks prior the patients’ death,

• health care and professionals support in the last months/weeks prior the patients’ death,

• informal caregivers‘ informal/family support during the last months/weeks prior patients death,

 recent challenges and bereavement,

• future prospects, concerns and wishes.

Needs for bereavement support will be addressed as well, since this type of interview is ethically challenging. Contact to local counselling agencies and self-help groups will be provided for bereavement support, if needed.

##### Serial interviews with family physicians

Family physicians will be interviewed face-to-face shortly after the patient or patient/caregiver interview at each study point. A short semi-structured interview guide will cover the family physicians’ perspectives of their patients’ and informal caregivers’ needs, available services and barriers to the provision of care (i.e. collaboration with medical specialists, nursing facilities, hospitals. Main topics addressed in the baseline interviews with family physicians are:

• significance and challenges in health care and end-of-life care for frail elderly people,

• health care/support needs of frail older patients’ towards the end of life,

• family/informal caregivers’ role and support needs,

• health care practice and cooperation needs,

• family practitioners’ and other health professionals roles and responsibilities,

• future prospects and suggestions to improve health care and end of life care for frail older patients.

The interview guide was tested for comprehensibility and handling in pilot interviews with two family practitioners not taking part in the study.

Follow-up interviews will mainly focus on changes in the respective patients’ illness/frailty trajectory, needs and provision of care (i.e. occurrence of crisis, need for hospital admission, and involvement of additional health professionals and/or services). If a patient dies, his/her family physician will be invited for an additional interview covering topics similar to the bereavement interviews (see above).

##### Data recording and memos

All interviews will be digitally recorded for transcription with the permission of the interviewees. The researchers will make comprehensive field notes to facilitate contextualisation of the data. Therefore postscripts, interview and operational memos will provide additional information on the context and course of the interviews.

#### Phase 2: focus group discussions

Focus group discussions will be held with healthcare professionals experienced in end-of-life care for elderly people. The discussions will be moderated by two experienced researchers. The group discussions will be structured using a topic guide. Each group begins with a round of introductions including information about participants’ age, location and practical experience, followed by a presentation and in-depth discussion of the major findings of phase 1. Participants need to consent to audio recording and full transcription of the discussions.

#### Phase 3: expert workshop

In a one-day expert workshop, major results will be presented and discussed using interactive techniques (i.e. small group work, meta-plan technique). Key themes derived from phase 1 and 2 will be presented and discussed to develop recommendations for practice. The participants will outline strategies for improving care for frail older people towards the end of life. The workshop will be recorded for documentation. The participants will receive a summary with main themes and recommendations with an invitation to give additional comments after the workshop.

### Data management and quality assurance

Recorded interviews as well as groups discussions will be fully transcribed verbatim. Transcription by a research assistant familiar with transcription of verbal data will cover paralinguistic features, such as voice tone/specific intonation or pauses. To ascertain high quality, all transcripts will be proof-checked with the interview audio-file by the interviewer.

The software programme MAXQDA 10 will be used for data management, coding and subsequent qualitative data analysis [44,45]. It also supports the retrieval of segments of coded text across a number of different interviews or field notes for comparison and contrasting.

Initial interviews will be reflected and discussed in the research team to review the interviewer behaviour, thoroughly review the interview guides, and ascertain overall quality of data collection and processing. The same applies to the critical reflection upon the focus group discussions in the second phase of the study. In the on-going research process, each interview and group discussion will be coded by two researchers in order to reduce bias and to improve the validity of the results. Analysis workshops will take place to regularly review the analytic process and evolving themes.

In addition, researchers will be supervised and coached by an external supervisor, to offer a forum for reflection on emotionally draining themes [46], which might arise in this project when confronted with (increasing) dependency and care needs, family dynamics, transition into dying phase, loss, and grief [23].

### Data analysis

Corresponding to the different types of qualitative data generated in the different phases of the study several approaches of qualitative data analysis will be used, primarily grounded theory and narrative analysis.

#### Phase 1: longitudinal patient-centred case studies

Analysis of the individual interviews and field notes will start soon after the first interview and take place alongside the on-going data collection in order to include and further develop emerging themes and first key concepts in the subsequent interviews [29]. Serial interviews are analysed using an inductive approach according to principles of grounded theory [25,42] and narrative analysis [26,27]. Data analysis and interpretation will take place on different levels covering comparative cross-sectional and longitudinal analysis within and across cases and target groups.

The analytical process of the serial interviews will start with reading, sequencing the interview text(s) into meaningful segments (thematic fields), and inductive coding in an iterative process. Codes will be generated to unfurl the data and to identify possible “hidden meanings” [42,43]. Microanalysis of key interview sequences will support this further. Special attention will be directed at initial interview sequences and narratives conveyed by the interviewees in the course of the interview [47]. For narrative analysis, a pluralistic approach was chosen drawing on the narratives’ context, structure, and content [26,27].

The emergent codes serve to stimulate the expansion, transformation and re-conceptualisation of the gathered information. Researchers’ field notes, interview postscripts and interview memos will enhance the interpretation. The codes will be subsumed to categories and subcategories. Key concepts of the literature, including the researchers’ previous own work in the field and the work of our collaborators, serve as sensitizing concepts for the development of basic descriptive categories. During the coding process, constant comparison and continuous writing of code and (provisional) theoretical memos will foster the analysis and development of key concepts.

The interview data will be analysed separately for each target group (patients, informal caregivers, family physicians). Subsequent comparative analysis will contrast the different views within and across these groups, and highlight emerging themes, convergence and divergence of perspectives. Emerging themes will be tested by marshalling the evidence from all data to support or refute them.

For longitudinal analysis, data will be organised as patient/informal caregiver/family physician sets. For single case reconstruction and typecast, additional steps of data analysis will use approaches of biographical research in social sciences [48], health services research [30], and narrative inquiry on chronic illness [28]. This narrative approach allows analysis of chronological aspects, i.e. how the different elements are sequenced, how the past shapes perceptions of the present and vice versa, and how both shape perceptions of the future [49].

#### Phase 2: focus group discussions

For data analysis of focus group discussions a qualitative descriptive approach and theme analysis will combine inductive and deductive coding strategies. Theme analysis and qualitative description will stay relatively close to the data. They are appropriate to focus the experiences and perceptions of professionals in health care, and allow to first insights into the informants’ views of a particular theme [50]. Principles of socio-linguistic conversation analysis and documentary method [51] will be applied, – based on the reconstruction of the sequential order of the discussion, to draw special attention to sequences in which participants show a high commitment (i.e. overlapping contributions of several participants) or in periods of silence within the group discussion.

In order to find out differences and similarities regarding the perceptions of health professionals, comparative analyses within and across groups will be carried out (i.e. comparing the family practitioner group and specialist palliative care group).

#### Phase 3: expert workshop

Thematic analysis will condense the expert group discussions during the final workshop. After the workshop, participants will receive a draft of the condensed main themes and recommendations and a request for additional comments in order to validate the results.

## Discussion

Internationally and nationally, there is a recent intensive debate on health care needs in older age, as well as emerging efforts to facilitate primary palliative care to alleviate the end of life of patients with non-malignant life limiting illness or advanced frailty [5,17,32,40,41]. In this study, the health care related needs and experiences of frail elderly patients as well as their informal caregivers and family physicians will be analysed. To our knowledge, this is the first prospective, qualitative study on end of life care for frail elderly people in Germany.

Results will contribute to the understanding of patients’ and informal caregivers’ needs and perceptions in unstable and downward phases of the illness/frailty trajectory and their changes over time, and provide information on how these needs and expectations are currently met, and which barriers may exist in the health care system. Findings can inform stakeholders and policy makers in health service organisation, and end-of-life care as well as practitioners, and yield an evidence base for recommendations to improve health care for older patients with increasing frailty taking into account concepts and priorities of concerned parties (patients, family, nurses, physicians). Results and recommendations can then be studied further in implementation research projects. The interdisciplinary expert workshop will be a first milestone for dissemination of the results beyond scientific publication.

### Limitations and challenges

There are a number of challenges and limitations to the study which stem from the complex qualitative design and the sensitivity of the subject [23,39,41,46,52]. Incomplete data in terms of perspectives from the patients and informal caregivers must be expected due to aggravation of illness/frailty or death. Major concerns address informed consent [39,46], confidentiality and anonymity in this sensitive and emotionally charged context. The potential impacts on both the researched and researchers must be considered [53].

Our qualitative study based on relatively small numbers of cases does not claim to be generalizable in a statistical sense. Instead our focus is primarily on providing contextualised in-depth insights on the evolution of support needed from family practitioners in downward phases of the illness/frailty trajectory based on the comparison of multiple perspectives.

## Competing interests

The authors declare that they have no conflicts of interest.

## Authors’ contributions

GMM and NSCH drafted the manuscript. NSCH, JB and EHP conceived the study and are responsible for field access and supervision of the research team. GMM, KG and KK recruit the participants, carry out the interviews and analyse the qualitative data. SP gives specialist advice in qualitative analysis, nursing and ethics. All authors read and approved the final paper.

## Pre-publication history

The pre-publication history for this paper can be accessed here:

http://www.biomedcentral.com/1471-2296/14/52/prepub
